# An Evolution Gaining Momentum—The Growing Role of Artificial Intelligence in the Diagnosis and Treatment of Spinal Diseases

**DOI:** 10.3390/diagnostics12040836

**Published:** 2022-03-29

**Authors:** Andre Wirries, Florian Geiger, Ludwig Oberkircher, Samir Jabari

**Affiliations:** 1 Spine Center, Hessing Foundation, Hessingstrasse 17, 86199 Augsburg, Germany; andre.wirries@hessing-stiftung.de; 2 Center of Orthopaedic and Trauma Surgery, Philipps University of Marburg, Baldingerstrasse, 35043 Marburg, Germany; oberkirc@med.uni-marburg.de; 3 Neuropathological Institute, University Hospitals Erlangen, Schwabachanlage 6, 91054 Erlangen, Germany; samir.jabari@fau.de

**Keywords:** artificial intelligence, machine learning, deep learning, spine, supervised learning, unsupervised learning, review

## Abstract

In recent years, applications using artificial intelligence have been gaining importance in the diagnosis and treatment of spinal diseases. In our review, we describe the basic features of artificial intelligence which are currently applied in the field of spine diagnosis and treatment, and we provide an orientation of the recent technical developments and their applications. Furthermore, we point out the possible limitations and challenges in dealing with such technological advances. Despite the momentary limitations in practical application, artificial intelligence is gaining ground in the field of spine treatment. As an applying physician, it is therefore necessary to engage with it in order to benefit from those advances in the interest of the patient and to prevent these applications being misused by non-medical partners.

## 1. Introduction

Artificial-intelligence-based technologies are increasingly entering the everyday medical treatment of spinal disorders. The influence on therapy decisions, on structures of health care systems and insurance companies, as well as the associated industry have the potential—with all benefits and drawbacks—to fundamentally change medical practice. Although the term artificial intelligence, as it is used today, was defined as early as 1956 [1], it is necessary to understand what these technologies can do today and where the limits of their implementation lie. These limits should be kept in mind in order to ensure reliable application.

For emerging technologies such as artificial intelligence, the Gartner hype cycle describes five successive development phases, starting with a technological trigger, moving through a rapidly reached peak of expectations into a valley of disappointment, followed by a slower process called the path of enlightenment, before reaching a plateau of productivity [2]. The technological trigger can be seen in the combination of massive advances in computing power and the increasing availability of big data in recent years, which has enabled a form of detailed data exploitation and processing that was unimaginable just a few years ago. In terms of spinal treatments, we are in the subsequent phase of greatly increased general awareness, and on the way to a peak of expectations. This phase is characterized by an exponential increase in scientific interest in these topics. Currently, under the search terms “artificial intelligence” and “spine”, 37% of all publications on Pubmed to date were indexed in 2020 and 2021 alone.

As with any emerging technology, new possibilities should not be underestimated in terms of their potential pitfalls. These lead to failures and setbacks in the course of time and thus establish the next development phase, as described by the Gartner hype cycle, characterized by disappointments and fading attention. On closer inspection, corresponding issues and pitfalls that may form a basis for these future disappointments are already emerging today and should not be neglected. This phase of declining interest is followed by a slower qualitative development until an actual productive use is established. It can be assumed that the application of artificial intelligence in the treatment of spinal diseases will also be subject to these processes. Accordingly, one should be aware of both the potentials and the challenges of these current developments in order to understand them and be able to address them properly.

## 2. Typical Forms of Artificial Intelligence Currently Applied in Spine Care

### 2.1. Artificial Intelligence, Machine Learning, and Neural Network

Artificial intelligence (AI) is a broad generic term. It encompasses various forms of automated decision making. One of the increasingly important forms in medical applications is machine learning. Simply, varying input data is used to predict an outcome through its assessment at decision points. Within this context, machine learning is mostly described as the decision-making process itself, commonly used in random forest algorithms or decision trees. Nowadays, a typical application of machine learning applies data and neural networks, trying to mimic the human brain. In these cases, the individual decision points in a neural network correspond to the functions of neurons in a biological brain. The rules used at decision points can range from simple yes/no decisions to the most complex mathematical formulations. The number of communicating decision points, each with its own set of rules, is not limited. An array of multiple decision points is therefore referred to as an artificial neural network (ANN).

### 2.2. Neural Networks and the “Black Box”

If several artificial neural decision levels are connected, then a deep neural network is created. Repeated decision making in such a neural network with constant comparison of an actual result with the one predicted in the network is called training. This mimics a learning process—hence the machine learning term. In this process, each decision-making procedure is followed by a correction mechanism that enables fine-tuning of the neural network and thus an optimized prediction. The process is called backpropagation, and is based on algorithms that have been developed since the 1980s [3]. The overall process within a deep neural network is called deep learning.

It is understandable that decision making in artificial deep neural networks can reach a high complexity. It becomes difficult to explain at first glance, in terms of which results are produced and how the decision is achieved. This establishes the myth of the “black box”, based solely on the complexity of decision generation. Ultimately, however, all machine learning is comprehensible and analyzable, albeit sometimes at great expense. The basis of result generation remains conditioned by the mathematical framework and cannot override it. Up to a certain point, this succeeds in imitating human learning processes, but to this day it remains an imitation.

### 2.3. Major Forms of Machine Learning Applied in Spine Care

The totality in which AI applications, and machine learning in particular, are constructed and applied today has been presented several times, including the detailed review by Galbusera et al. from 2019 [4]. The application of machine learning in medicine and especially in the treatment of spinal diseases can be described based on the form of the learning process, distinguishing three basic forms, discussed in the following subsections.

#### 2.3.1. Supervised Learning

**Figure 1:**
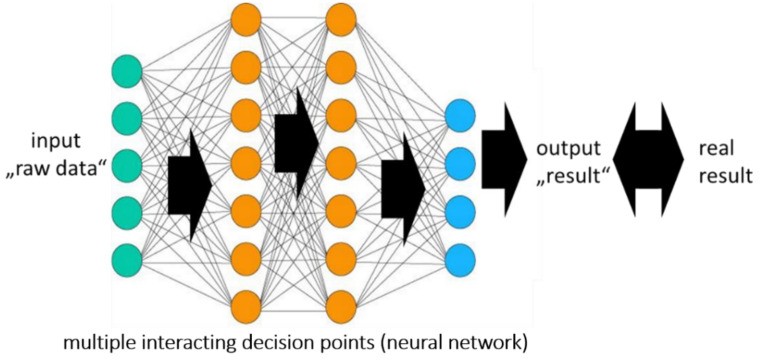
Supervised learning using a neural network.

In supervised learning, the prediction of an already known result is learned using training datasets. The goal is to find the relationship between an initial dataset and the corresponding result. The prediction is optimized by minimizing the difference between the predicted and the real result (Figure 1). This difference is described as “loss of function”. Its minimization leads to a usable prediction, which can then be applied to comparable datasets with unknown results. The number of datasets necessary to establish a good prediction depends substantially on the complexity of the problem and the quality of the data.

#### 2.3.2. Reinforcement Learning

A special form of supervised learning can be seen in reinforcement learning. In this application, the known results are missing. Instead, the produced result is evaluated externally, and a correction of the result production is carried out thereupon.

#### 2.3.3. Unsupervised Learning

**Figure 2:**
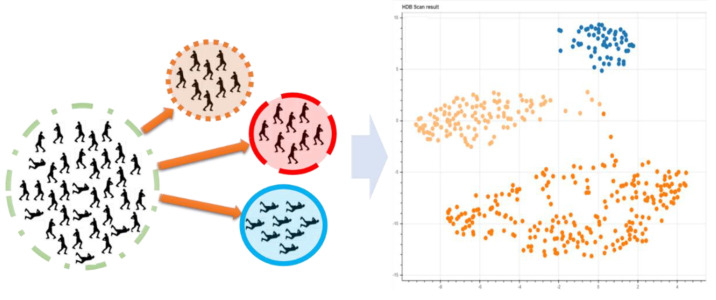
In unsupervised learning, patterns are searched for in a set of data and applied to form data clusters.

Unsupervised learning means that datasets are analyzed without any pre-known results. However, the goal here is not the prediction of a specific result, but the recognition of patterns, groupings, and features, with the aim of extracting new information from the existing data (Figure 2). In the applications published today, it is mainly big data analyses that make use of this form and lead to new insights.

## 3. AI Applications in the Diagnosis and Treatment of Spinal Disorders

We performed an extensive analysis of PubMed-listed publications using the search terms “spine, spinal, lumbar, thoracic, cervical, machine learning, deep learning, supervised learning, unsupervised learning, artificial intelligence”. A total of 1144 publications, published since 1990, were identified. Of these, 866 (76.7%) were published in the last 10 years and 420 have been published since 2020 alone (37.2%). Of these 420 publications, the following exclusion criteria applied: not subject to peer review, not related to the topic of spine, represented meta-analyses, reviews, or case reports. This left 167 publications in 2020 and 2021 that could be attributed to the topic of artificial intelligence in the treatment of spinal disorders. With 102 publications, radiological applications and imaging represented the majority of AI applications in this field.

### 3.1. Diagnostic Imaging

Due to the close connection of the field of radiology with technological advances, the application of artificial intelligence has established itself in this area at an early stage.

Thus, the recognition of spinal structures and consequently the measurement of parameters can be automated [5,6,7]. In this context, increasingly better algorithms are being published, so that automated analyses of spine images are now possible, and the detection of classical pathologies has already been realized as an inevitable further development [8,9,10,11]. The application of convolutional neural networks (CNN), that are based on an imitation of visual cortical data processing, are only one part of the overall concept [12]. In recent years, it has become more and more possible to achieve evaluations and classifications of image data that go beyond the known level of quality [6,13,14,15]. The correlation of image data with underlying systemic diseases is yet another step that has already been made to some extent today [10,16,17,18]. The accuracy and efficiency already achieved by the application of advanced algorithms in the detection and interpretation of spinal imaging data are game-changing progressions, considering the importance of this imaging in treatment decision making, and will have a long-term impact on the care of spinal disorders [19].

### 3.2. Robotics

With the improvement of image data processing also comes an optimized application of navigation in spine surgery. With these prerequisites, the application of robot-assisted spine surgery has also entered the market in recent years with the aim of optimizing surgical outcomes, minimizing risks, and reducing the invasiveness of procedures. To date, several surgical robots have been approved for human application, the four most common being the Excelsius GPS Robot from Globus Medical, the SpineAssist from MAZOR Robotics in Israel, the ROSA from Medtech in France, and the Da Vinci Surgical System from Intuitive Surgical in the USA. All have in common that their implementation in the field of spine surgery is still slowly taking place, in some cases only in larger case series. Jonathon J. Rasouli provides a detailed overview of the updated results regarding the accuracy of pedicle screw positioning, radiation exposure, and operation duration [20]. Applicability is now no longer an issue; however, the current advantages of the application are still rather small compared with a well-trained spine surgeon.

### 3.3. Biomechanical Spine Assessement

By rapidly developing the ability to analyze image data in recent years, the application of AI to analyze gait and movement patterns and identify pathological abnormalities in spinal disorders is also becoming feasible [12,21,22]. Estimating biomechanical variables such as load and strain on the spine and predicting what loads will occur and how they can ideally be mitigated will allow therapies to be tailored to the patient’s individual biomechanical conditions in the future [23,24,25].

### 3.4. Prediction of Diagnosis and Outcome

A rapid increase in AI applications has been seen in recent years in the field of diagnosis and prognosis of spine disorders. With an increasing general understanding of machine learning in the medical community, its applications in the field of spine medicine are increasingly noticed as well. Earlier attempts have been able to demonstrate its applicability [26,27,28,29,30], but it is the use of modern machine learning algorithms that have made it possible to raise prognosis and diagnosis of spinal diseases to a competitive level [31,32].

Currently published applications have made it increasingly possible to answer previously scientifically unresolved questions. For example, Ames was already able to show in 2019 (with the European and International Spine Study Groups) that a reliable grouping of risk clusters for adult deformity surgery is possible [33,34,35]. The prediction qualities and applicability of this are continuously improved [36]. In the meantime, similar approaches have been presented in several publications. Thus, models for predicting outcome after surgical or conservative treatment of disc herniation have been described as well as the probability of a recurrence of disc herniation [37,38,39,40]. Particularly for the prediction of precisely specified complications and outcomes under well-defined conditions, the currently available algorithms can achieve excellent prediction values in some cases. Examples include the prediction of the outcome after surgical therapy of intramedullary tumors, the probability of C5 paresis after certain types of cervical spine surgery, the prediction of major complications after spinal fusion, or the probability of recurrence after osteomyelitis [17,41,42,43,44,45,46,47,48,49]. Accordingly, the frequency of such publications, some of which have excellent predictive capabilities, is increasing [50].

In the meantime, for broader questions—such as automated differentiation of pathologies—there are still relatively insufficient results for a real application [7,9,31,51]. However, even here it is only a matter of time until the available data quality allows optimal prediction quality.

## 4. AI in Spine Care Future

### 4.1. Data Quality and Verification

Following the old saying, “rubbish in–rubbish out”, even the best-programmed AI application cannot pass the test of reality if the original data with which it was trained is flawed [19]. The technical capability to work with personal data and to use it economically developed in the last two decades prior to the awareness that this data and its collection must be subject to supervision. As a result, we now live in an era of uncontrolled data collection with inadequate control mechanisms. 

Additionally, as a result of the growing need for data collection, continuous patient evaluation and re-evaluation is needed. That may lead to the patient feeling overwhelmed, affecting their interest and willingness to cooperate. This may negatively impact the quality of the collected data causing misleading results.

Accordingly, it can be observed in medically relevant areas that the quality of data does not always meet the standard needed for the establishment of artificial intelligence algorithms. For the treatment of spinal diseases, this means that internal and external control of algorithms is necessary. An important step would be developing an extension of the existing guidelines for study protocols to pair with the requirements of establishing AI applications, as has been or is currently being carried out for most guidelines [52,53,54]. Although AI-based predictive algorithms can be complex, they should be judged by at least the same standards as traditional research models to assure a healthy development of these techniques [55].

### 4.2. Database Repositories for Use in Spine Therapy

In the field of therapy of spinal diseases, the establishment of large data collections is still in its infancy. As far as the authors know, the SORG group (“Sorg Orthopaedic Re-search Group”) has established several online available data collections, partly with freely available prediction models in the field of spine surgery [44,56]. Apart from that, the Austrian Spinal Cord Injury Study [57], as well as the national North American and British spine registries, with their respective societies, have established relevant and available repositories [58,59]. However, apart from the SORG group, these are hardly externally validated [60]. The above-mentioned repositories and those currently being established (mostly national spine registries) are still far away from a regular application and validation, as it is now common in other medical fields [61].

### 4.3. Advantages of AI in the Future of Spine Diagnostics and Treatment

The application of AI-based predictions and models will change the approach to spinal pathologies. In hardly any other medical field has it been so difficult to assess the success and necessity of interventions. The correct indication is one of the most important basic requirements for successful spine therapy. The implementation of artificial intelligence in the treatment of spinal diseases will increasingly change this in the coming years. However, its real impact on improving the quality of care is still to be seen. The transformation from a diagnosis-based decision to a combined decision, based on diagnosis and individual patient data, will only result in optimized care if machine learning can successfully and verifiably deliver the right results. In the hands of a physician as an assistance tool, patients could then benefit from faster, customized care [23,62,63,64]. As a result, it may be possible to avoid costly, still unsuccessful, treatments, as well as the chronicity of spinal conditions, by integrating AI applications into clinical practice.

### 4.4. Ethical Issues of AI in Spine Diagnostics and Treatment

The reduction in unnecessary costs can also have negative effects, as political interests do not necessarily serve the best interests of patients. If individual costs become transparent, then an economical and often a political decision to avoid high individual costs is inevitable. AI applications in spine therapy should therefore remain in the hands of treating physicians as a supporting tool in decision making. Misinterpretation of such algorithms, for example by insurance companies, should be avoided at all costs. Algorithms for predicting therapy success or failure should not be misunderstood and misused as a cost regulator [65,66,67,68,69]. 

Establishments of AI algorithms as decision support tools for medical care have proven in prominent examples that discrimination and misinterpretation can occur. The Framingham Heart Study, for example, led to predictions with massive racial discrimination because the underlying data were not sufficiently validated in this respect [70]. Similar problems can be expected in the application in the field of spinal therapy [71]. The main cause is the transfer of a human bias into the training data used to build an AI algorithm. If this bias is not recognized during the original data generation, then it will be reflected in the AI predictions in a highly veiled form. As a result, a corresponding bias may be barely detectable, if at all, in the context of the final AI application. For example, it was recently shown that an AI algorithm for detecting pathologies in chest radiographs developed a neglect for those pathologies in certain populations that were characterized by poor medical care during data collection [72].

Therefore, AI will become a tool, but it will never be a replacement. Even the most audacious fantasies of the future always see AI as an instrument. According to the medical principle that “there is nothing that does not exist”, an authority will always be required to control, question, and correct the application of algorithms. In the future, this authority will have to be in the hands of the physician who knows their patient population personally, can assess it, and who is able to understand whether the medical care is really providing the expected help to their individual patient or not [73].

## 5. Conclusions

We are at a turning point in the care of spine diseases. In the next few years, whether we like it or not, a standard will be established that will be based on predictions and diagnoses utilizing AI algorithms. On the one hand, until then, a bumpy road of development is to be expected, and setbacks should be anticipated with a watchful eye to avoid putting patients at risk. On the other hand, physicians should be actively involved in the development of AI algorithms to ensure that these tools remain in their hands and will not be overtaken by medically unoriented insurance companies and politicians.

## References

[1] McCarthy J., Minsky M.L., Rochester N., Shannon C.E. (2006). A proposal for the dartmouth summer research project on artificial intelligence, 31 August 1955. AI Mag..

[2] Fenn J., LeHong H. (2011). Hype Cycle for Emerging Technologies.

[3] Rumelhart D.E., Hinton G.E., Williams R.J. (1986). Learning representations by back-propagating errors. Nature.

[4] Galbusera F., Casaroli G., Bassani T. (2019). Artificial intelligence and machine learning in spine research. JOR Spine.

[5] Yang D., Xiong T., Xu D., Zhou S.K., Xu Z., Chen M., Park J., Grbic S., Tran T.D., Chin S.P. (2019). Deep Image-To-Image Recurrent Network with Shape Basis for Automatic Vertebra Labeling in Large-Scale 3D CT Volumes. U.S. Patent.

[6] Galbusera F., Niemeyer F., Wilke H.-J., Bassani T., Casaroli G., Anania C., Costa F., Brayda-Bruno M., Sconfienza L.M. (2019). Fully automated radiological analysis of spinal disorders and deformities: A deep learning approach. Eur. Spine J..

[7] Meng N., Cheung J.P.Y., Wong K.K., Dokos S., Li S., Choy R.W., To S., Li R.J., Zhang T. (2022). An artificial intelligence powered platform for auto-analyses of spine alignment irrespective of image quality with prospective validation. EClinicalMedicine.

[8] Guermazi A., Tannoury C., Kompel A.J., Murakami A.M., Ducarouge A., Gillibert A., Li X., Tournier A., Lahoud Y., Jarraya M. (2021). Improving radiographic fracture recognition performance and efficiency using artificial intelligence. Radiology.

[9] Galbusera F., Cina A., Bassani T., Panico M., Sconfienza L.M. (2021). Automatic diagnosis of spinal disorders on radiographic images: Leveraging existing unstructured datasets with natural language processing. Glob. Spine J..

[10] Chianca V., Cuocolo R., Gitto S., Albano D., Merli I., Badalyan J., Cortese M.C., Messina C., Luzzati A., Parafioriti A. (2021). Radiomic machine learning classifiers in spine bone tumors: A multi-software, multi-scanner study. Eur. J. Radiol..

[11] Karandikar P., Massaad E., Hadzipasic M., Kiapour A., Joshi R.S., Shankar G.M., Shin J.H. (2022). Machine learning applications of surgical imaging for the diagnosis and treatment of spine disorders: Current state of the art. Neurosurgery.

[12] Liu Y., Sui X., Liu C., Kuang X., Hu Y. (2019). Automatic lumbar spine tracking based on siamese convolutional network. J. Digit. Imaging.

[13] Yang H.S., Kim K.R., Kim S., Park J.Y. (2021). Deep learning application in spinal implant identification. Spine.

[14] Niemeyer F., Galbusera F., Tao Y., Kienle A., Beer M., Wilke H.-J. (2021). A deep learning model for the accurate and reliable classification of disc degeneration based on MRI data. Investig. Radiol..

[15] Lafage R., Ang B., Alshabab B.S., Elysee J., Lovecchio F., Weissman K., Kim H.J., Schwab F., Lafage V. (2021). Predictive model for selection of upper treated vertebra using a machine learning approach. World Neurosurg..

[16] Ulivieri F.M., Rinaudo L., Messina C., Piodi L.P., Capra D., Lupi B., Meneguzzo C., Sconfienza L.M., Sardanelli F., Giustina A. (2021). Bone strain index predicts fragility fracture in osteoporotic women: An artificial intelligence-based study. Eur. Radiol. Exp..

[17] Kim J., Ryu H., Kim S.W., Oh J.K., Kim T.H. (2021). Prediction of recurrence in pyogenic vertebral osteomyelitis by artificial neural network using time-series data of c-reactive protein: A retrospective cohort study of 704 patients. Spine.

[18] de Vries B.C.S., Hegeman J.H., Nijmeijer W., Geerdink J., Seifert C., Groothuis-Oudshoorn C.G.M. (2021). Comparing three machine learning approaches to design a risk assessment tool for future fractures: Predicting a subsequent major osteoporotic fracture in fracture patients with osteopenia and osteoporosis. Osteoporos. Int..

[19] Hornung A.L., Hornung C.M., Mallow G.M., Barajas J.N., Espinoza Orias A.A., Galbusera F., Wilke H.J., Colman M., Phillips F.M., An H.S. (2022). Artificial intelligence and spine imaging: Limitations, regulatory issues and future direction. Eur. Spine J..

[20] Rasouli J.J., Shao J., Neifert S., Gibbs W.N., Habboub G., Steinmetz M.P., Benzel E., Mroz T.E. (2021). Artificial intelligence and robotics in spine surgery. Glob. Spine J..

[21] Jiang N., Luk K.D.-K., Hu Y. (2017). A machine learning-based surface electromyography topography evaluation for prognostic prediction of functional restoration rehabilitation in chronic low back pain. Spine.

[22] Zhang J., Lockhart T.E., Soangra R. (2014). Classifying lower extremity muscle fatigue during walking using machine learning and inertial sensors. Ann. Biomed. Eng..

[23] Xie N., Wilson P.J., Reddy R. (2022). Use of machine learning to model surgical decision-making in lumbar spine surgery. Eur. Spine J..

[24] Lin M., Abd M.A., Taing A., Tsai C.T., Vrionis F.D., Engeberg E.D. (2021). Robotic replica of a human spine uses soft magnetic sensor array to forecast intervertebral loads and posture after surgery. Sensors.

[25] Aghazadeh F., Arjmand N., Nasrabadi A. (2020). Coupled artificial neural networks to estimate 3D whole-body posture, lumbosacral moments, and spinal loads during load-handling activities. J. Biomech..

[26] van Hooff M.L., van Loon J., van Limbeek J., de Kleuver M. (2014). The nijmegen decision tool for chronic low back pain. Development of a clinical decision tool for secondary or tertiary spine care specialists. PLoS ONE.

[27] Mann N.H., Brown M.D. (1991). Artificial intelligence in the diagnosis of low back pain. Orthop. Clin. N. Am..

[28] Coupé V.M., van Hooff M.L., de Kleuver M., Steyerberg E.W., Ostelo R.W. (2016). Decision support tools in low back pain. Best Pract. Res. Clin. Rheumatol..

[29] McGirt M.J., Sivaganesan A., Asher A.L., Devin C.J. (2015). Prediction model for outcome after low-back surgery: Individualized likelihood of complication, hospital readmission, return to work, and 12-month improvement in functional disability. Neurosurg. Focus.

[30] Lee M.J., Cizik A.M., Hamilton D., Chapman J.R. (2014). Predicting surgical site infection after spine surgery: A validated model using a prospective surgical registry. Spine J..

[31] Mallow G.M., Siyaji Z.K., Galbusera F., Espinoza-Orias A.A., Giers M., Lundberg H., Ames C., Karppinen J., Louie P.K., Phillips F.M. (2021). Intelligence-based spine care model: A new era of research and clinical decision-making. Glob. Spine J..

[32] Gutman M.J., Schroeder G.D., Murphy H., Flanders A.E., Vaccaro A.R. (2021). Artificial intelligence in spine care. Clin. Spine Surg..

[33] Durand W.M., Daniels A.H., Hamilton D.K., Passias P., Kim H.J., Protopsaltis T., LaFage V., Smith J.S., Shaffrey C., Gupta M. (2020). Artificial intelligence models predict operative versus nonoperative management of patients with adult spinal deformity with 86% accuracy. World Neurosurg..

[34] Ames C.P., Smith J.S., Pellisé F., Kelly M., Alanay A., Acaroglu E., Pérez-Grueso F.J.S., Kleinstück F., Obeid I., Vila-Casademunt A. (2019). Artificial intelligence based hierarchical clustering of patient types and intervention categories in adult spinal deformity surgery: Towards a new classification scheme that predicts quality and value. Spine.

[35] Durand W.M., Lafage R., Hamilton D.K., Passias P.G., Kim H.J., Protopsaltis T., Lafage V., Smith J.S., Shaffrey C., Gupta M. (2021). Artificial intelligence clustering of adult spinal deformity sagittal plane morphology predicts surgical characteristics, alignment, and outcomes. Eur. Spine J..

[36] Joshi R.S., Lau D., Ames C.P. (2021). Artificial intelligence for adult spinal deformity: Current state and future directions. Spine J..

[37] Harada G.K., Siyaji Z.K., Mallow G.M., Hornung A.L., Hassan F., Basques B.A., Mohammed H.A., Sayari A.J., Samartzis D., An H.S. (2021). Artificial intelligence predicts disk re-herniation following lumbar microdiscectomy: Development of the “RAD” risk profile. Eur. Spine J..

[38] Wirries A., Geiger F., Hammad A., Oberkircher L., Blumcke I., Jabari S. (2020). Artificial intelligence facilitates decision-making in the treatment of lumbar disc herniations. Eur. Spine J..

[39] Staartjes V.E., Quddusi A., Klukowska A.M., Schröder M.L. (2020). Initial classification of low back and leg pain based on objective functional testing: A pilot study of machine learning applied to diagnostics. Eur. Spine J..

[40] Pedersen C.F., Andersen M.O., Carreon L.Y., Eiskjaer S. (2020). Applied machine learning for spine surgeons: Predicting outcome for patients undergoing treatment for lumbar disc herniation using PRO data. Glob. Spine J..

[41] Massaad E., Ha Y., Shankar G.M., Shin J.H. (2022). Clinical prediction modeling in intramedullary spinal tumor surgery. Acta Neurochir. Suppl..

[42] Wang K.Y., Suresh K.V., Puvanesarajah V., Raad M., Margalit A., Jain A. (2021). Using Predictive Modeling and Machine Learning to Identify Patients Appropriate for Outpatient Anterior Cervical Fusion and Discectomy. Spine.

[43] Wang H., Tang Z.R., Li W., Fan T., Zhao J., Kang M., Dong R., Qu Y. (2021). Prediction of the risk of C5 palsy after posterior laminectomy and fusion with cervical myelopathy using a support vector machine: An analysis of 184 consecutive patients. J. Orthop. Surg. Res..

[44] Shah A.A., Devana S.K., Lee C., Bugarin A., Lord E.L., Shamie A.N., Park D.Y., van der Schaar M., SooHoo N.F. (2021). Prediction of major complications and readmission after lumbar spinal fusion: A machine learning-driven approach. World Neurosurg..

[45] Pasha S., Shah S., Newton P. (2021). Machine learning predicts the 3d outcomes of adolescent idiopathic scoliosis surgery using patient-surgeon specific parameters. Spine.

[46] Martini M.L., Neifert S.N., Oermann E.K., Gilligan J.T., Rothrock R.J., Yuk F.J., Gal J.S., Nistal D.A., Caridi J.M. (2021). Application of cooperative game theory principles to interpret machine learning models of nonhome discharge following spine surgery. Spine.

[47] Kuris E.O., Veeramani A., McDonald C.L., DiSilvestro K.J., Zhang A.S., Cohen E.M., Daniels A.H. (2021). Predicting readmission after anterior, posterior, and posterior interbody lumbar spinal fusion: A neural network machine learning approach. World Neurosurg..

[48] Khan O., Badhiwala J.H., Akbar M.A., Fehlings M.G. (2021). Prediction of worse functional status after surgery for degenerative cervical myelopathy: A machine learning approach. Neurosurgery.

[49] Karhade A.V., Bongers M.E.R., Groot O.Q., Cha T.D., Doorly T.P., Fogel H.A., Hershman S.H., Tobert D.G., Srivastava S.D., Bono C.M. (2021). Development of machine learning and natural language processing algorithms for preoperative prediction and automated identification of intraoperative vascular injury in anterior lumbar spine surgery. Spine J..

[50] Ogink P.T., Groot O.Q., Karhade A.V., Bongers M.E.R., Oner F.C., Verlaan J.J., Schwab J.H. (2021). Wide range of applications for machine-learning prediction models in orthopedic surgical outcome: A systematic review. Acta Orthop..

[51] Wirries A., Geiger F., Hammad A., Redder A., Oberkircher L., Ruchholtz S., Bluemcke I., Jabari S. (2021). Combined artificial intelligence approaches analyzing 1000 conservative patients with back pain-a methodological pathway to predicting treatment efficacy and diagnostic groups. Diagnostics.

[52] Ibrahim H., Liu X., Denniston A.K. (2021). Reporting guidelines for artificial intelligence in healthcare research. Clin. Exp. Ophthalmol..

[53] Kitaguchi D., Watanabe Y., Madani A., Hashimoto D.A., Meireles O.R., Takeshita N., Mori K., Ito M. (2021). Artificial intelligence for computer vision in surgery: A call for developing reporting guidelines. Ann. Surg..

[54] Liu X., Cruz Rivera S., Moher D., Calvert M.J., Denniston A.K. (2020). Reporting guidelines for clinical trial reports for interventions involving artificial intelligence: The CONSORT-AI extension. Lancet Digit. Health.

[55] Bazoukis G., Hall J., Loscalzo J., Antman E.M., Fuster V., Armoundas A.A. (2022). The inclusion of augmented intelligence in medicine: A framework for successful implementation. Cell Rep. Med..

[56] Shah A.A., Karhade A.V., Park H.Y., Sheppard W.L., Macyszyn L.J., Everson R.G., Shamie A.N., Park D.Y., Schwab J.H., Hornicek F.J. (2021). Updated external validation of the SORG machine learning algorithms for prediction of ninety-day and one-year mortality after surgery for spinal metastasis. Spine J..

[57] Aschauer-Wallner S., Mattiassich G., Aigner L., Resch H. (2017). The austrian spinal cord injury study: A registry for patients living with a traumatic spinal cord injury. Spinal Cord Ser. Cases.

[58] Sebastian A.S. (2016). Database research in spine surgery. Clin. Spine Surg..

[59] Asher A.L., Knightly J., Mummaneni P.V., Alvi M.A., McGirt M.J., Yolcu Y.U., Chan A.K., Glassman S.D., Foley K.T., Slotkin J.R. (2020). Quality outcomes database spine care project 2012–2020: Milestones achieved in a collaborative North American outcomes registry to advance value-based spine care and evolution to the American spine registry. Neurosurg. Focus.

[60] Groot O.Q., Bindels B.J.J., Ogink P.T., Kapoor N.D., Twining P.K., Collins A.K., Bongers M.E.R., Lans A., Oosterhoff J.H.F., Karhade A.V. (2021). Availability and reporting quality of external validations of machine-learning prediction models with orthopedic surgical outcomes: A systematic review. Acta Orthop..

[61] Saravi B., Hassel F., Ülkümen S., Zink A., Shavlokhova V., Couillard-Despres S., Boeker M., Obid P., Lang G.M. (2022). Artificial intelligence-driven prediction modeling and decision making in spine surgery using hybrid machine learning models. J. Pers. Med..

[62] Cerrato P., Halamka J. (2021). The Digital Reconstruction of Healthcare: Transitioning from Brick and Mortar to Virtual Care.

[63] Etzel C.M., Veeramani A., Zhang A.S., McDonald C.L., DiSilvestro K.J., Cohen E.M., Daniels A.H. (2021). Supervised machine learning for predicting length of stay after lumbar arthrodesis: A comprehensive artificial intelligence approach. J. Am. Acad. Orthop. Surg..

[64] Berjano P., Langella F., Ventriglia L., Compagnone D., Barletta P., Huber D., Mangili F., Licandro G., Galbusera F., Cina A. (2021). The influence of baseline clinical status and surgical strategy on early good to excellent result in spinal lumbar arthrodesis: A machine learning approach. J. Pers. Med..

[65] Wiens J., Saria S., Sendak M., Ghassemi M., Liu V.X., Doshi-Velez F., Jung K., Heller K., Kale D., Saeed M. (2019). Do no harm: A roadmap for responsible machine learning for health care. Nat. Med..

[66] Kedra J., Gossec L. (2020). Big data and artificial intelligence: Will they change our practice?. Jt. Bone Spine.

[67] Chan A.K., Santacatterina M., Pennicooke B., Shahrestani S., Ballatori A.M., Orrico K.O., Burke J.F., Manley G.T., Tarapore P.E., Huang M.C. (2020). Does state malpractice environment affect outcomes following spinal fusions? A robust statistical and machine learning analysis of 549,775 discharges following spinal fusion surgery in the United States. Neurosurg. Focus.

[68] Lee M.S., Grabowski M.M., Habboub G., Mroz T.E. (2020). The impact of artificial intelligence on quality and safety. Glob. Spine J..

[69] Cartolovni A., Tomicic A., Lazic Mosler E. (2022). Ethical, legal, and social considerations of AI-based medical decision-support tools: A scoping review. Int. J. Med. Inform..

[70] Gijsberts C.M., Groenewegen K.A., Hoefer I.E., Eijkemans M.J., Asselbergs F.W., Anderson T.J., Britton A.R., Dekker J.M., Engström G., Evans G.W. (2015). Race/ethnic differences in the associations of the framingham risk factors with carotid IMT and cardiovascular events. PLoS ONE.

[71] Char D.S., Shah N.H., Magnus D. (2018). Implementing machine learning in health care—Addressing ethical challenges. N. Engl. J. Med..

[72] Seyyed-Kalantari L., Zhang H., McDermott M., Chen I.Y., Ghassemi M. (2021). Underdiagnosis bias of artificial intelligence algorithms applied to chest radiographs in under-served patient populations. Nat. Med..

[73] James C.A., Wachter R.M., Woolliscroft J.O. (2022). Preparing clinicians for a clinical world influenced by artificial intelligence. JAMA.

